# Cryobiopsy Outperforms Fine-Needle Aspiration for Mediastinal Lymphadenopathy: A Within-Patient Diagnostic Accuracy Study and Clinical Predictors of Success

**DOI:** 10.3390/jcm15145656

**Published:** 2026-07-19

**Authors:** María Hidalgo, Carlos Carpio, Sergio Alcolea, Pablo Mariscal, Maria Alejandra Castillo, Ana Rodriguez, Isabel Esteban, Pilar López, Rodolfo Álvarez-Sala

**Affiliations:** 1 Department of Pulmonology, La Paz–Cantoblanco–Carlos III University Hospital, Faculty of Medicine, UAM, IdiPAZ, 28046 Madrid, Spain; mhidalgosanchez@salud.madrid.org (M.H.); sergio.alcolea@salud.madrid.org (S.A.); pablo.mariscal@salud.madrid.org (P.M.); rodolfo.alvarezsala@salud.madrid.org (R.Á.-S.); 2 Department of Pulmonology, Nuestra Señora de Sonsoles Hospital, 05004 Ávila, Spain; mariacastillo93@gmail.com; 3 Department of Pathology, La Paz-Cantoblanco-Carlos III University Hospital, IdiPAZ, 28046 Madrid, Spain; anamargarita.rodriguez@salud.madrid.org (A.R.); misabel.esteban@salud.madrid.org (I.E.); plferrez@salud.madrid.org (P.L.)

**Keywords:** mediastinal cryobiopsy, transbronchial needle aspiration, endobronchial ultrasound, mediastinal lymphadenopathy

## Abstract

**Background/Objectives**: Accurate diagnosis of lymph node pathology is essential for the management of both malignant and benign diseases. This study compares the diagnostic performance of endobronchial ultrasound-guided transbronchial mediastinal cryobiopsy and endobronchial ultrasound-guided transbronchial needle aspiration (EBUS-TBNA) in a real-world cohort. **Methods**: A single-center retrospective observational study was conducted, including 91 patients who underwent both cryobiopsy and EBUS-TBNA for lymph node evaluation. EBUS-TBNA was performed with a 19-G needle and cryobiopsy with a 1.1 mm flexible cryoprobe. Clinical characteristics, positron emission tomography–computed tomography (PET–CT) findings, ultrasonographic characteristics of the lymph nodes, diagnostic yield, and procedure-related complications were analyzed. Diagnostic accuracy, sensitivity, specificity, and factors associated with correct diagnosis were assessed. **Results**: The median age of the cohort was 65 years [interquartile range-IQR-56–74], 63.7% were male, and 74.7% were former or current smokers. Malignant disease was ultimately diagnosed in 60 patients (65.9%), of whom 78.3% had non-lymphoproliferative tumours and 21.7% had lymphoproliferative malignancies. The diagnostic accuracy of cryobiopsy was 89%, compared with 82.4% for EBUS-TBNA. The paired comparison between the two techniques did not reach statistical significance. After exclusion of non-diagnostic samples, sensitivity was numerically higher for cryobiopsy (96.4%, 95% CI 90.7–100) than for EBUS-TBNA (88.9%, 95% CI 79.6–98.2). Factors associated with correct diagnosis included chronic obstructive pulmonary disease for cryobiopsy, cardiovascular comorbidities, and maximum SUV for EBUS-TBNA. In malignant lesions, cryobiopsy showed higher diagnostic accuracy than EBUS-TBNA (90% vs. 80% accuracy), particularly in lymphoproliferative diseases. In benign lesions, both techniques agreed in 17 (54%) of cases, with cryobiopsy showing a higher yield for sarcoidosis and contributing to the diagnosis of tuberculosis. Complications were infrequent and minor, with no major adverse events reported. **Conclusions**: Cryobiopsy showed numerically higher diagnostic accuracy and sensitivity compared with EBUS-TBNA, particularly in malignant and lymphoproliferative disorders, with a favorable safety profile. These findings support its role as a valuable complement—or alternative—to conventional EBUS-TBNA in the assessment of mediastinal lymph nodes.

## 1. Introduction

Endobronchial ultrasound-guided transbronchial needle aspiration (EBUS-TBNA) is currently the technique of choice for the evaluation of mediastinal lymphadenopathy. It is a minimally invasive, outpatient procedure that has progressively replaced mediastinoscopy [1]. Accordingly, European and American societies recommend EBUS-TBNA as the initial approach in the assessment of mediastinal pathology [1,2]. Despite its excellent diagnostic performance in non-small cell lung cancer, with sensitivity reaching 92% and specificity reaching 100% [3], the amount of tissue obtained can occasionally be limited and insufficient for certain conditions such as rare tumours, lymphomas, or benign diseases including sarcoidosis and tuberculosis [4,5]. For example, the diagnostic yield of EBUS-TBNA in sarcoidosis is relatively modest, ranging from 54% to 93%, with a pooled performance of 79% [6]. In the case of lymphomas, sensitivity is around 66% at best [7]. As a result, many patients require more invasive diagnostic procedures such as mediastinoscopy to obtain a definitive diagnosis.

Mediastinal cryobiopsy was first introduced in 2021, when Zhang et al. [8] reported a diagnostic yield of 53% for benign lesions and 25% for extrapulmonary tumours using EBUS-TBNA, compared with 80.9% and 91.7%, respectively, using nodal cryobiopsy. Cryobiopsy obtains specimens with better-preserved architecture when compared with EBUS-TBNA samples, which may enhance diagnostic and staging accuracy for malignant mediastinal lesions—particularly in cases with suspected lymphoproliferative disorders or when a second EBUS is necessary due to insufficient initial sampling. Additionally, it appears to improve diagnostic performance in granulomatous diseases and may allow for expanded molecular profiling in lung cancer [8]. Cryobiopsy might serve as an intermediate step between EBUS-TBNA and surgical mediastinoscopy, which may reduce the need for the latter in a significant proportion of cases.

To date, only one study has compared two tunnelling strategies for EBUS-TBNA prior to cryobiopsy using 22-G and 19-G needles, with no increase in diagnostic yield [9]. Most comparative studies rely on a 22-G TBNA needle [10], and only a few centres use a needle-knife to facilitate mucosal incision [7]. Our study is among the few with a relatively large number of EBUS-TBNA procedures performed exclusively using a 19-G needle, which may enable a more balanced comparison with the 22-G needle, as it involves comparing a histological rather than a cytological needle with a lymph node biopsy sample. Additionally, there are no mediastinal cryobiopsy studies that specifically evaluate cardiovascular or respiratory comorbidities as variables associated with diagnostic yield outcomes. Finally, nodal ultrasound features have been extensively investigated as predictors of malignancy in the context of conventional EBUS-TBNA; however, their role as predictive factors of diagnostic success in mediastinal cryobiopsy has not yet been specifically evaluated, nor has the relationship with PET-CT, which has only recently been addressed as a patient selection criterion rather than a predictor of improvement in the diagnostic rate [11].

For these reasons, the aim of this study was to compare the diagnostic yield and diagnostic accuracy of EBUS-TBNA using a 19-G needle and mediastinal cryobiopsy, with both techniques performed in the same patient and at the same nodal station during a single procedure, allowing an intra-patient comparison. Additionally, we aimed to identify clinical, radiological, and sonographic factors associated with diagnostic performance.

## 2. Materials and Methods

### 2.1. Study Design

This was a single-center retrospective observational study including consecutive patients who underwent endobronchial ultrasound-guided transbronchial needle aspiration (EBUS-TBNA) and transbronchial mediastinal cryobiopsy as part of routine clinical practice between January 2024 and January 2025. The clinical procedures were performed according to standard clinical indications and were not carried out for research purposes. The primary objective of the study was to evaluate the diagnostic yield of mediastinal cryobiopsy across different pathologies, compare it with that of EBUS-TBNA, and assess the safety of the procedure. The retrospective review, analysis, and publication of clinical data generated during routine care were approved by the Research Ethics Committee of La Paz University Hospital (PI-6764; date of approval: 8 August 2025). Data extraction, database preparation, statistical analysis, and manuscript preparation were performed after Ethics Committee approval. The study was conducted in accordance with the principles of the Declaration of Helsinki. All patients were thoroughly informed about the clinical procedure, and written informed consent was obtained for the performance of the procedure as part of standard clinical practice.

Each patient served as their own control, as both EBUS-TBNA and subsequent cryobiopsy were performed during the same procedure. EBUS-TBNA samples were sent for cytological analysis, whereas nodal cryobiopsy specimens were submitted for histopathological examination. Whenever an infection was suspected, the sample was submitted to the Microbiology Department. Rapid on-site evaluation (ROSE) was not performed due to its unavailability at our institution.

### 2.2. Reference Standard

The reference standard was defined as the final diagnosis established after completion of the diagnostic work-up, integrating histopathological, microbiological, clinical, and radiological data.

For non-lymphoproliferative tumours, the diagnosis was based on histopathological confirmation obtained from EBUS-TBNA, cryobiopsy, or additional sampling techniques when required (including endobronchial biopsy or CT-guided fine-needle aspiration), and validated through multidisciplinary consensus in a tumour board.

For lymphoproliferative malignancies, the diagnosis required histological evaluation with immunophenotypic characterization, supported by additional procedures such as bone marrow biopsy when indicated, and confirmed in a dedicated hematology multidisciplinary board.

Tuberculosis was diagnosed based on microbiological confirmation (positive culture from bronchial aspirate or bronchoalveolar lavage), in conjunction with compatible clinical and radiological findings.

Sarcoidosis was diagnosed based on compatible clinical and radiological findings together with histological evidence of non-necrotizing granulomas, after exclusion of alternative causes, and confirmed through multidisciplinary discussion in an interstitial lung disease team.

For benign conditions, the diagnosis was supported by clinical and radiological follow-up over a minimum of 12 months, including chest CT imaging confirming stability or resolution of lymphadenopathy.

### 2.3. Patients

Patients aged over 18 years with mediastinal lymphadenopathy greater than 10 mm in size were included. Patients underwent sampling of the same nodal station using both techniques during a single endoscopic procedure (EBUS-TBNA followed by cryobiopsy), which allowed a direct comparison of the results obtained since each patient served as their own control.

A fluorodeoxyglucose positron emission tomography (FDG-PET) scan or a thoracic computed tomography (CT) scan prior to the procedure was needed.

Exclusion criteria were coagulopathy, an endoscopic or anaesthetic contraindication due to high procedural risk, terminal illness, haemodynamic instability, severe respiratory failure, or refusal to undergo the procedure by the patient.

As part of routine clinical practice, all patients had a recent (within three months) blood test including full blood count, biochemistry, and coagulation profile, as well as a resting electrocardiogram.

The following variables were recorded: demographic characteristics, smoking status, and relevant comorbidities. Cardiovascular risk factors included diabetes mellitus, dyslipidemia, and systemic hypertension. Also, the standardized uptake value (SUV) on FDG-PET/CT was recorded, along with nodal station samples and their sonographic characteristics, including size, homogeneity, vascularization, and morphology. In addition, the number of needle passes and cryobiopsies performed was documented, as well as any procedure-related complications, including bleeding, infection, and pneumomediastinum. The final diagnosis (malignancy or non-malignancy) was also collected, and cases of malignant diseases were classified as non-lymphoproliferative or lymphoproliferative tumours.

### 2.4. Procedure

All procedures were performed by a pulmonologist experienced in EBUS-TBNA. Deep sedation was used in all cases, with a laryngeal mask airway. Anaesthetic management was provided entirely by the Anaesthesiology Department, with continuous monitoring of oxygen saturation, blood pressure, heart rate, respiratory rate, and electrocardiogram.

A linear EBUS bronchoscope (BF-UC190F, Olympus^®^, Tokyo, Japan) was introduced through the laryngeal mask [12]. Once the target lymphadenopathy was located, it was punctured using a 19-G Olympus ViziShot 2 Flex^®^ needle, and two to four needle aspirations were performed. Samples were sent to the pathologist in a cell fixative medium (Cytolyt^®^), and also to the Microbiology Department in sterile saline when infection was suspected.

Subsequently, through the same needle tract, a 1.1 mm cryoprobe connected to an ERBECRYO^®^ 2 unit (Erbe^®^ Elektromedizin, GmbH, Tübingen, Germany) was introduced, and between three and five cryobiopsies were obtained, with a freezing time of six seconds. Samples were placed in formalin for histopathological analysis and in sterile saline for microbiological studies when infection was considered.

All patients underwent post-procedure chest radiography to rule out pneumothorax or pneumomediastinum. Patients were discharged home 2–3 h after the procedure, once complications had been excluded.

### 2.5. Statistical Analysis

Quantitative variables were expressed as the mean and standard deviation or as the median and interquartile range [IQR], depending on their distribution. Categorical variables were summarized as absolute frequencies and percentages. A global descriptive analysis of the entire cohort was performed, followed by stratified analyses according to diagnostic groups: neoplastic disease (non-lymphoproliferative tumours and lymphoproliferative malignancies) and benign pathology. Factors associated with a correct diagnosis were evaluated separately for each technique using the chi-square test or Fisher’s exact test for categorical variables, and the Mann–Whitney U test for non-parametric continuous variables. Given the within-patient study design, these analyses were conducted independently for each technique (EBUS-TBNA and cryobiopsy), and no unpaired statistical tests were used to directly compare diagnostic performance between them. For paired comparisons of diagnostic accuracy between EBUS-TBNA and cryobiopsy within the same patients, McNemar’s test was used. Diagnostic performance was assessed using measures of diagnostic yield, diagnostic accuracy, sensitivity, and specificity as appropriate. Diagnostic yield was defined as the proportion of procedures providing sufficient material for diagnosis, whereas diagnostic accuracy was defined as the proportion of correct diagnoses according to the reference standard. Sensitivity and specificity were calculated for the distinction between neoplastic and benign/non-neoplastic disease using evaluable diagnostic samples only. Non-diagnostic results were excluded from sensitivity and specificity calculations because no interpretable diagnostic classification was available, but were included as diagnostic failures in diagnostic yield and diagnostic accuracy analyses. All statistical tests were two-sided, and a *p*-value < 0.05 was considered statistically significant. Statistical analyses were conducted using R version 4.3.3 (R Foundation for Statistical Computing, Vienna, Austria).

## 3. Results

### 3.1. Study Population

A total of 91 patients were included. The median age was 65 years [56–74]. Of the total cohort, 58 patients (63.7%) were male, and 68 (74.7%) were former or current smokers. Regarding comorbidities, cardiovascular risk factors were present in 49 patients (53.8%), while respiratory disease was documented in 35 patients (38.5%) (Table 1).

With respect to the lymph node stations sampled, 60 (65.9%) patients had lymph nodes measuring ≥1.5 cm, and the infracarinal station was sampled in 42 (46.2%) cases. On PET-CT, the median SUV was 9.4 [5.7–12.5], with increased uptake observed in 46 of the 60 patients with available PET-CT data (76.7%) (Table 2).

Of all patients included, 60 (65.9%) had malignant disease, and 31 (34.1%) had benign pathology. Among those with malignancies, 47 patients (78.3%) had non-lymphoproliferative tumours, while 13 (21.7%) had lymphoproliferative ones (Table 3).

### 3.2. Diagnostic Yield

#### 3.2.1. Overall Diagnostic Yield

Cryobiopsy and EBUS-TBNA provided sufficient material for diagnosis in 86 (94.5%) and 82 (90.1%) patients, respectively (Table 4). The diagnostic accuracy of cryobiopsy was 89% (81/91), whereas that of EBUS-TBNA was 82.4% (75/91).

Both techniques agreed on the classification of malignant or benign disease in 72 patients (79.1%). Discordant results were observed in 12 cases: in 9 patients (9.9%), cryobiopsy achieved a correct diagnosis, whereas EBUS-TBNA did not, while the opposite situation occurred in 3 patients (3.3%). The difference did not reach statistical significance according to McNemar’s test (*p* = 0.146).

After excluding non-diagnostic samples, the sensitivity of EBUS-TBNA for distinguishing neoplastic from benign/non-neoplastic disease was 88.9% (48/54; 95% confidence interval [CI], 79.6–98.2), with a specificity of 96.4% (27/28; 95% CI, 81.7–99.9). Cryobiopsy showed a sensitivity of 96.4% (54/56; 95% CI, 90.7–100) and a specificity of 96.4% (27/28; 95% CI, 81.7–99.9). Because non-diagnostic samples were excluded separately for each technique, sensitivity and specificity estimates were calculated using technique-specific evaluable denominators. Non-diagnostic results were observed in nine EBUS-TBNA procedures and seven cryobiopsy procedures and were reported separately in the contingency table. The corresponding diagnostic performance table, including true positives, false positives, true negatives, false negatives, and non-diagnostic results, is shown in Table 5.

Factors associated with diagnostic accuracy for cryobiopsy included COPD (*p* = 0.019). For EBUS-TBNA, factors associated with diagnostic accuracy were cardiovascular comorbidities (*p* = 0.046) and maximum SUV on PET-CT (*p* = 0.038) (Table 6).

Regarding the number of samples obtained per patient, the median was two [2,3] for cryobiopsies and four [3,4] for EBUS-TBNA. When considering only cases with a correct diagnosis, the median number of samples was four [3,4] for cryobiopsy and two [2,3] for EBUS-TBNA.

#### 3.2.2. Diagnostic Yield in Malignant Lesions

A total of 60 patients were ultimately diagnosed with malignant neoplasia. Cryobiopsy established a correct diagnosis in 54 cases (90%), whereas EBUS-TBNA did so in 48 (80%). Cryobiopsy correctly identified malignant disease in 10% more patients compared with EBUS-TBNA. Among the six patients in whom cryobiopsy failed to provide a correct diagnosis, histological lymph node samples were obtained in two cases (3.3%) but did not demonstrate malignancy. Similarly, this occurred in six patients (10%) in the EBUS-TBNA group (Table 5).

Both techniques agreed on a correct diagnosis in 47 patients (78.3%). Cryobiopsy achieved a correct diagnosis in seven patients (11.7%) in whom EBUS-TBNA did not, whereas a correct diagnosis was obtained exclusively by EBUS-TBNA in only one patient (1.7%).

Regarding non-lymphoproliferative tumours, both techniques failed to achieve a correct diagnosis in three patients (6.4%). In the remaining 44 patients (93.6%), both procedures yielded concordant results. (Table 4).

Lymphoproliferative malignancies were identified in 13 patients. In four cases (30.8%), both techniques established a correct diagnosis, while in three patients (23.1%), neither technique achieved a correct diagnosis. Cryobiopsy established a correct diagnosis in six patients (46.1%) who were not correctly diagnosed by EBUS-TBNA, which failed to achieve a correct diagnosis in nine cases (Table 4).

#### 3.2.3. Diagnostic Yield in Benign Lesions

A total of 31 patients were ultimately diagnosed with benign disease after completion of the diagnostic work-up. Both techniques agreed on a correct diagnosis in 17 patients (54%). Sarcoidosis was diagnosed in seven patients (70%) by cryobiopsy and in one patient (10%) by EBUS-TBNA. There were two cases of tuberculosis, both of which were diagnosed based on microbiological cultures obtained from bronchial aspirate or bronchoalveolar lavage; cryobiopsy samples showed granulomatous inflammation consistent with tuberculous lymphadenitis, with additional positive cultures from these samples (Table 4).

### 3.3. Complications

No major complications were observed during or immediately after the cryobiopsy procedure or in the subsequent four weeks. Minor adverse events included five cases of mild bleeding, two cases of moderate bleeding, and one case of mild, self-limiting pneumomediastinum. No cases of pneumothorax or mediastinitis were recorded.

## 4. Discussion

Cryobiopsy showed a numerically higher diagnostic accuracy than EBUS-TBNA in our cohort, although the paired comparison did not reach statistical significance; this difference was particularly apparent in patients with lymphoproliferative malignancies and benign disorders. After exclusion of non-diagnostic samples, cryobiopsy also showed numerically higher sensitivity for distinguishing neoplastic from benign/non-neoplastic disease, whereas specificity was high and identical for both techniques. Moreover, our study identifies, to our knowledge for the first time, specific clinical factors associated with achieving a correct diagnosis for both cryobiopsy and EBUS-TBNA, highlighting the potential value of patient baseline characteristics in guiding the choice of sampling technique.

The reported diagnostic yield of mediastinal cryobiopsy ranges from 60% to 100%, whereas that of EBUS-TBNA ranges from 54% to 87%. Our results are consistent with these previously described ranges [13,14,15], even in the absence of ROSE. In a systematic review and meta-analysis by Sehgal et al. [16], ROSE did not improve diagnostic yield or shorten procedure time during TBNA; however, it was associated with fewer needle passes and a lower need for additional bronchoscopic procedures to reach a definitive diagnosis. These findings are also supported by pathology-based evidence, suggesting that ROSE enhances lesion targeting and enables preliminary diagnosis, thereby potentially reducing the need for further interventions without increasing procedural time or complications during EBUS-TBNA [17].

Regarding malignant aetiology, in this study, the diagnostic results for non-lymphoproliferative tumours were concordant between EBUS-TBNA and cryobiopsy in 44 of 47 cases, although no differentiation by histological subtype was made. These findings closely match previously published data, which report a diagnostic accuracy of 94% for cryobiopsy compared to approximately 87% for EBUS-TBNA [18,19]. Other studies have indicated that, in the case of rare pulmonary tumours, the diagnostic yield of cryobiopsy appears to be significantly higher than that of TBNA, with reported rates of 90% and nearly 58%, respectively [20,21]. This is clinically relevant, as it may reduce the number of mediastinoscopies that would otherwise be performed in patients with negative EBUS-TBNA results, with subsequent reductions in healthcare costs, operating room utilization, waiting times, and surgery-related complications.

In lymphoproliferative diseases, cryobiopsy demonstrated a diagnostic accuracy of 76.9% (10 out of 13 cases in our study) compared to 30.8% for EBUS-TBNA. Mediastinoscopy and core needle biopsy are typically the preferred techniques for diagnosing lymphoma. Certain lymphoma subtypes, such as marginal zone and follicular lymphomas, are more difficult to diagnose via EBUS-TBNA due to the limited tissue volume and the focal nature of the sampling within a single nodal area. In this context, cryobiopsy represents a promising alternative, potentially avoiding more invasive and costly diagnostic procedures. Reported diagnostic yields for cryobiopsy in lymphomas range from 80% to 100%. Nevertheless, published case series on cryobiopsy in this setting are relatively small, with no more than 40 cases included in the largest series, highlighting the need for further research in this field [22,23].

Cardiovascular risk factors—such as hypertension, dyslipidemia, and diabetes—may act as predictors of higher diagnostic accuracy, potentially through their association with pulmonary carcinogenesis and lymph node inflammatory changes, which increase the likelihood of positive EBUS-TBNA findings. However, inflammatory remodeling may lead to fibrosis and tissue induration, which can hinder needle penetration and increase the risk of false-negative results [24]. Moreover, chronic respiratory diseases are closely linked to tobacco use, and smoking induces histopathologic and biologic alterations in the bronchial mucosa—including chronic inflammation, remodeling, and accumulation of carbonaceous material—that may obscure lesions and contribute to false negatives. Consequently, multiple or larger specimens with well-preserved architecture may be required, which may explain the numerically higher diagnostic accuracy observed with cryobiopsy in patients with both malignant and benign chronic respiratory diseases. Nevertheless, a potential confounding factor should be considered: the majority of smokers also exhibit other cardiovascular risk factors, which may further influence these associations.

For benign conditions, including sarcoidosis and tuberculosis, cryobiopsy has demonstrated a higher diagnostic performance, with previously reported diagnostic yields ranging from 78% to 100%, compared with 53–100% for EBUS-TBNA [25,26]. In our cohort, there were ten cases of sarcoidosis and two of tuberculosis. The diagnostic yield of cryobiopsy for sarcoidosis was 70%, compared to 10% for EBUS-TBNA. In the case of tuberculosis, both cases were diagnosed based on microbiological cultures obtained from bronchial aspirate or bronchoalveolar lavage, in accordance with the predefined reference standard, while cryobiopsy samples showed granulomatous inflammation and yielded additional positive cultures, resulting in correct diagnoses in both cases despite the small sample size.

Our study has several notable strengths. First, extensive sampling was achieved across almost all lymph node stations, with the exception of station 2L, enhancing the representativeness of our findings. Second, EBUS-TBNA was performed using 19-G histology needles, whereas most previous studies have relied on cytology needles; this approach may allow for a more appropriate comparison with cryobiopsy specimens, as both provide histological samples. Additionally, we explored potential associations between diagnostic accuracy and lymph node ultrasound characteristics, as well as PET/CT metabolic activity—variables that have not been systematically evaluated in prior studies. Furthermore, we investigated the relationship between cardiovascular risk factors and chronic respiratory disease and diagnostic accuracy for both EBUS-TBNA and mediastinal cryobiopsy, offering novel insights into clinical factors that may influence procedural performance.

This study has several limitations that should be acknowledged. First, we did not record the additional procedural time required to perform mediastinal cryobiopsy, nor did we evaluate its cost-effectiveness. These aspects may have important implications for clinical workflows and resource allocation, but they fell outside the primary objective of assessing diagnostic accuracy. Second, the absence of rapid on-site evaluation (ROSE) in our bronchoscopy suite may have influenced sample adequacy, potentially increasing the number of needle passes or prolonging the procedure. Additionally, although each patient served as their own control, operator expertise and subtle procedural variations may still have affected performance outcomes. Third, for some cases, the reference standard incorporated information obtained during the diagnostic work-up, including pathological findings from the index procedures, EBUS-TBNA and cryobiopsy, together with clinical, radiological, and multidisciplinary assessment. Therefore, incorporation bias cannot be completely excluded, particularly in the small number of discordant cases lacking fully independent pathological confirmation, and may have resulted in a modest overestimation of diagnostic performance. Finally, the study was conducted at a single center with a relatively limited sample size for certain subgroups (particularly lymphoproliferative disorders), which may restrict the generalizability of our findings and warrant validation in larger, multicenter cohorts.

## 5. Conclusions

Based on our findings, mediastinal cryobiopsy emerges as a diagnostic technique with numerically higher diagnostic accuracy and sensitivity than EBUS-TBNA for the evaluation of mediastinal lymphadenopathy, while maintaining an excellent safety profile. In our cohort, cryobiopsy provided a higher proportion of correct benign and malignant diagnoses, with particularly meaningful added value in lymphoproliferative disorders, where the limitations of EBUS-TBNA were most pronounced. Taken together, these results support the integration of nodal cryobiopsy as a key diagnostic tool—either as a complementary method or, in selected scenarios, as an alternative to conventional EBUS-TBNA—in the assessment of mediastinal lymph nodes. Nevertheless, prospective multicenter studies with larger and more diverse patient populations are needed to confirm these findings and to better define the optimal role of cryobiopsy within diagnostic algorithms.

## Figures and Tables

**Table 1:** Characteristics of patients.

	Median [IQR] or *n* (%)
Males	58 (63.7%)
Age, years	65 [56–74]
Smoking	68 (74.7%)
Cardiovascular risk factors	49 (53.8%)
Chronic lung disease	35 (38.5%)
Dyslipidemia	31 (34.1%)
COPD	30 (33.0%)
Systemic hypertension	29 (31.9%)
Diabetes mellitus	12 (13.2%)
Obstructive sleep apnea	7 (7.7%)
Ischemic heart disease	5 (5.5%)
Asthma	3 (3.3%)
Atrial fibrillation	2 (2.2%)
Cerebrovascular disease	2 (2.2%)

Data expressed as median (interquartile range) or number (percentage). Cardiovascular risk factors: diabetes mellitus, dyslipidemia, systemic hypertension; chronic lung disease: asthma, chronic obstructive pulmonary disease, obstructive sleep apnea; COPD: chronic obstructive pulmonary disease.

**Table 2:** Characteristics of the studied lymph node stations.

	*n* (%)
Lymph node size	
8–10 mm	5 (5.5%)
10–12 mm	3 (3.3%)
13–15 mm	23 (25.3%)
16–19 mm	12 (13.2%)
20–30 mm	28 (30.8%)
>30 mm	20 (22.0%)
Lymph node size categorized by size	
≤1.5 cm	31 (34.1%)
>1.5 cm	60 (65.9%)
Lymph node region analyzed categorized	
Paratracheal (2R, 4R, 4L)	20 (22%)
Subcarinal (7)	42 (46.2%)
Hilar (10R, 10L, 11R, 11L)	29 (31.9%)
Lymph node morphology	
Poorly defined	36 (39.6%)
Rounded	34 (37.4%)
Ovoid	13 (14.3%)
Triangular	8 (8.8%)
Lymph node homogeneity	
Heterogeneous	55 (60.4%)
Homogeneous	36 (39.6%)
Lymph node vascularization	
Mild	59 (64.8%)
Moderate	15 (16.5%)
Intense	17 (18.7%)
PET uptake	
Mild	1 (1.7%)
Moderate	13 (21.7%)
Intense	46 (76.7%)

Data expressed as number (percentage). PET: Positron emission tomography. PET-CT data were available for 60 patients; percentages for PET uptake are calculated based on this subgroup.

**Table 3:** Definitive diagnoses in included patients.

Diagnosis	*n* (%)
Non-lymphoproliferative tumour	47 (51.6%)
Adenocarcinoma	21 (23.1%)
Squamous cell carcinoma	9 (9.9%)
Small cell carcinoma	7 (7.7%)
Undifferentiated carcinoma	6 (6.6%)
Sarcomatoid carcinoma	2 (2.2%)
Melanoma	1 (1.1%)
Neuroendocrine carcinoma	1 (1.1%)
Lymphoproliferative tumour *	13 (14.3%)
Sarcoidosis †	10 (11%)
Pneumoconiosis	9 (9.9%)
Normal lymph node	6 (6.6%)
Reactive lymphoid hyperplasia ‡	4 (4.4%)
Tuberculosis	2 (2.2%)

Data expressed as number (percentage). * Lymphoproliferative tumours included lymphoma and one case of plasmocytoma. † Sarcoidosis corresponds to cases showing non-necrotizing granulomatous inflammation for which the final multidisciplinary diagnosis was sarcoidosis. ‡ Reactive lymphoid hyperplasia refers to benign reactive lymphoid proliferation without evidence of malignancy, granulomatous disease, or specific infection.

**Table 4:** Diagnostic results of endobronchial ultrasound-guided transbronchial needle aspiration (EBUS-TBNA) and lymph node cryobiopsy.

Diagnosis	EBUS-TBNA *n* (%)	Cryobiopsy *n* (%)	Definitive Diagnoses *n* (%)
Non-lymphoproliferative tumour	44 (48.4%)	44 (48.4%)	47 (51.6%)
Lymphoproliferative tumour	4 (4.4%)	10 (10.9%)	13 (14.3%)
Sarcoidosis	1 (1.1%)	7 (7.7%)	10 (11%)
Pneumoconiosis	7 (7.7%)	6 (6.6%)	9 (9.9%)
Normal lymph node	17 (18.7%)	10 (10.9%)	6 (6.6%)
Reactive lymphoid hyperplasia	9 (9.9%)	7 (7.7%)	4 (4.4%)
Tuberculosis	0 (0%)	2 (2.2%)	2 (2.2%)
Total diagnostic	82 (90.1%)	86 (94.5%)	91 (100%)

Data expressed as number (percentage). Percentages were calculated using the total study population (*n* = 91) as the denominator. EBUS-TBNA: Endobronchial ultrasound-guided transbronchial needle aspiration.

**Table 5:** Diagnostic performance of EBUS-TBNA and cryobiopsy for distinguishing neoplastic from benign/non-neoplastic disease according to the reference standard.

Technique	Result	Reference Standard Neoplastic	Reference Standard Benign/Non-Neoplastic	Total
EBUS-TBNA	Neoplastic	48	1	49
EBUS-TBNA	Non-neoplastic	6	27	33
EBUS-TBNA	Non-diagnostic	6	3	9
Cryobiopsy	Neoplastic	54	1	55
Cryobiopsy	Non-neoplastic	2	27	29
Cryobiopsy	Non-diagnostic	4	3	7

Sensitivity and specificity were calculated using evaluable diagnostic samples only, excluding non-diagnostic results, because these samples did not provide an interpretable diagnostic classification. Non-diagnostic samples were reported separately and were included as diagnostic failures in the diagnostic yield and diagnostic accuracy analyses. For EBUS-TBNA, sensitivity was 88.9% (48/54) and specificity was 96.4% (27/28). For cryobiopsy, sensitivity was 96.4% (54/56) and specificity was 96.4% (27/28). EBUS-TBNA: endobronchial ultrasound-guided transbronchial needle aspiration.

**Table 6:** Factors associated with a correct diagnosis by cryobiopsy and endobronchial ultrasound-guided transbronchial needle aspiration (EBUS-TBNA).

Variable	Category	Cryobiopsy *n* (%)	TBNA (%)	Cryobiopsy *p*	TBNA *p*
Sex	Male	52 (89.7%)	49 (84.5%)	0.794	0.493
	Female	29 (87.9%)	26 (78.8%)		
Smoking	Yes	63 (92.6%)	58 (85.3%)	0.057	0.215
	No	18 (78.3%)	17 (73.9%)		
Cardiovascular risk factors	Yes	45 (91.8%)	44 (89.8%)	0.352	0.046
	No	36 (85.7%)	31 (73.8%)		
Chronic lung disease	Yes	35 (100%)	30 (85.7%)	0.008	0.514
	No	46 (82.1%)	45 (80.4%)		
Systemic hypertension	Yes	25 (86.2%)	23 (79.3%)	0.559	0.594
	No	56 (90.3%)	52 (83.9%)		
Diabetes mellitus	Yes	10 (83.3%)	9 (75.0%)	0.500	0.469
	No	71 (89.9%)	66 (83.5%)		
Dyslipidemia	Yes	28 (90.3%)	27 (87.1%)	0.774	0.399
	No	53 (88.3%)	48 (80.0%)		
Ischemic heart disease	Yes	5 (100%)	5 (100%)	0.419	0.288
	No	76 (88.4%)	70 (81.4%)		
Atrial fibrillation	Yes	2 (100%)	2 (100%)	0.615	0.509
	No	79 (88.8%)	73 (82.0%)		
Cerebrovascular disease	Yes	2 (100%)	2 (100%)	0.615	0.509
	No	79 (88.8%)	73 (82.0%)		
Obstructive sleep apnoea	Yes	7 (100%)	5 (71.4%)	0.333	0.427
	No	74 (88.1%)	70 (83.3%)		
Asthma	Yes	3 (100%)	3 (100%)	0.536	0.416
	No	78 (88.6%)	72 (81.8%)		
COPD	Yes	30 (100%)	25 (83.3%)	0.019	0.872
	No	51 (83.6%)	50 (82.0%)		
Lymph node size > 1.5 cm	>1.5 cm	52 (86.7%)	48 (80.0%)	0.320	0.399
	≤1.5 cm	29 (93.5%)	27 (87.1%)		
Puncture territory	Paratracheal	19 (95.0%)	17 (85.0%)	0.545	0.668
	Infracarinal	36 (85.7%)	33 (78.6%)		
	Hilar	26 (89.7%)	25 (86.2%)		
Node morphology	Rounded	32 (94.1%)	28 (82.4%)	0.521	0.670
	Triangular	7 (87.5%)	7 (87.5%)		
	Ovoid	12 (92.3%)	12 (92.3%)		
	Poorly defined	30 (83.3%)	28 (77.8%)		
Node homogeneity	Homogeneous	31 (86.1%)	31 (86.1%)	0.474	0.454
	Heterogeneous	50 (90.9%)	44 (80.0%)		
Vascularisation	Moderate–intense	31 (96.9%)	25 (78.1%)	0.077	0.428
	Mild	50 (84.7%)	50 (84.7%)		
PET uptake	High	39 (84.8%)	34 (73.9%)	0.585	0.724
	Mild–moderate	11 (78.6%)	11 (78.6%)		
Clinical suspicion	Neoplasy	67 (90.5%)	60 (81.1%)	0.330	0.485
	Non-neoplasy	14 (82.4%)	15 (88.2%)		
SUV max	Correct diagnosis	9.4 [5.8–12.2]	10.8 [8.4–12.8]	0.571	0.038

Data expressed as median (interquartile range) or number (percentage). Cardiovascular risk factors: diabetes mellitus, dyslipidemia, systemic hypertension; chronic lung disease: asthma, chronic obstructive pulmonary disease, obstructive sleep apnea; COPD: chronic obstructive pulmonary disease; PET: positron emission tomography; SUV: standard of uptake value.

## Data Availability

The data presented in this study are not publicly available due to privacy and ethical restrictions related to patient confidentiality. De-identified data may be made available from the corresponding author upon reasonable request and subject to approval by the institutional ethics committee.

## Abbreviations

The following abbreviations are used in this manuscript:

AUC	Area Under the Curve
CI	Confidence Interval
COPD	Chronic Obstructive Pulmonary Disease
CT	Computed Tomography
EBUS	Endobronchial Ultrasound
EBUS-TBNA	Endobronchial Ultrasound-Guided Transbronchial Needle Aspiration
FDG	Fluorodeoxyglucose
FDG-PET	Fluorodeoxyglucose Positron Emission Tomography
G	Gauge
IQR	Interquartile Range
mm	Millimetres
PET-CT	Positron Emission Tomography–Computed Tomography
ROC	Receiver Operating Characteristic
ROSE	Rapid On-Site Evaluation
SUV	Standardized Uptake Value
TBNA	Transbronchial Needle Aspiration

## References

[1] Detterbeck F.C., Jantz M.A., Wallace M., Vansteenkiste J., Silvestri G.A., American College of Chest Physicians (2007). Invasive mediastinal staging of lung cancer: ACCP evidence-based clinical practice guidelines (2nd edition). CHEST.

[2] The American Thoracic Society and The European Respiratory Society (1997). Pretreatment Evaluation of Non-Small-Cell Lung Cancer. Am. J. Respir. Crit. Care Med..

[3] Herth F.J., Ernst A., Eberhardt R., Vilmann P., Dienemann H., Krasnik M. (2006). Endobronchial ultrasound-guided transbronchial needle aspiration of lymph nodes in the radiologically normal mediastinum. Eur. Respir. J..

[4] Eapen G.A., Shah A.M., Lei X., Jimenez C.A., Morice R.C., Yarmus L., Filner J., Ray C., Michaud G., Greenhill S.R. (2013). Complications, consequences, and practice patterns of endobronchial ultrasound-guided transbronchial needle aspiration: Results of the AQuIRE registry. Chest.

[5] Franke K.J., Bruckner C., Szyrach M., Ruhle K.H., Nilius G., Theegarten D. (2012). The contribution of endobronchial ultrasound-guided forceps biopsy in the diagnostic workup of unexplained mediastinal and hilar lymphadenopathy. Lung.

[6] Agarwal R., Srinivasan A., Aggarwal A.N., Gupta D. (2012). Efficacy and safety of convex probe EBUS-TBNA in sarcoidosis: A systematic review and meta-analysis. Respir. Med..

[7] Labarca G., Sierra-Ruiz M., Kheir F., Folch E., Majid A., Mehta H.J., Jantz M.A., Fernandez-Bussy S. (2019). Diagnostic accuracy of endobronchial ultrasound transbronchial needle aspiration in lymphoma. A systematic review and meta-analysis. Ann. Am. Thorac. Soc..

[8] Zhang J., Guo J.R., Huang Z.S., Fu W.L., Wu X.L., Wu N., Kuebler W.M., Herth F.J., Fan Y. (2021). Transbronchial mediastinal cryobiopsy in the diagnosis of mediastinal lesions: A randomised trial. Eur. Respir. J..

[9] Oruqaj G., Lohfink-Schumm S., Gattenloehner S., Pervizaj-Oruqaj L., Lo K., Krombach G., Seeger W., Grimminger F., Tello K., Hecker M. (2026). Diagnostic yield and histopathological assessment of EBUS-guided transbronchial mediastinal cryobiopsy: A prospective comparison of two tunnelling approaches. ERJ Open Res..

[10] Garcia Garcia M., Martinez Molina J., Salcedo Lobera E. (2025). Transbronchial mediastinal cryobiopsy guided by endobronchial ultrasonography: Case series of 234 cases. Eur. Respir. J..

[11] Todisco F., Patrucco F., Riberi L., Veljkovic A., Valsecchi L., Vallese G., Iovine P.R., Ubaldi M., Ielo G., Indellicati D. (2025). Practical application of radiological criteria to select patients undergoing cryo-EBUS. Eur. Respir. J..

[12] Hidalgo Sánchez M., Luján M., Alcolea Batres S., Álvarez Del Vayo J., Mariscal-Aguilar P., Carpio C., Álvarez-Sala Walther R. (2025). Evidence on Non-Invasive Respiratory Support During Flexible Bronchoscopy: A Narrative Review. J. Clin. Med..

[13] Botana-Rial M., Lojo-Rodríguez I., Leiro-Fernández V., Ramos-Hernández C., González-Montaos A., Pazos-Area L., Núñez-Delgado M., Fernández-Villar A. (2023). Is the diagnostic yield of mediastinal lymph node cryobiopsy (cryoEBUS) better for diagnosing mediastinal node involvement compared to endobronchial ultrasound-guided transbronchial needle aspiration (EBUS-TBNA)? A systematic review. Respir. Med..

[14] Fan Y., Zhang A.M., Wu X.L., Huang Z.S., Kontogianni K., Sun K., Fu W.-L., Wu N., Kuebler W.M., Herth F.J.F. (2023). Transbronchial needle aspiration combined with cryobiopsy in the diagnosis of mediastinal diseases: A multicentre, open-label, randomised trial. Lancet Respir. Med..

[15] Mathew R., Roy W.E., Thomas E.S., Meena N., Danilevskaya O. (2024). Meta-analysis and systematic review of mediastinal cryobiopsy versus endobronchial ultrasound-transbronchial needle aspiration (EBUS-TBNA) in the diagnosis of intrathoracic adenopathy. J. Thorac. Dis..

[16] Sehgal I.S., Dhooria S., Aggarwal A.N., Agarwal R. (2018). Impact of rapid on-site cytological evaluation (ROSE) on the diagnostic yield of transbronchial needle aspiration during mediastinal lymph node sampling: Systematic review and meta-analysis. Chest.

[17] Jain D., Allen T.C., Aisner D.L., Beasley M.B., Cagle P.T., Capelozzi V.L., Hariri L.P., Lantuejoul S., Miller R., Mino-Kenudson M. (2018). Rapid on-site evaluation of endobronchial ultrasound-guided transbronchial needle aspirations for the diagnosis of lung cancer: A perspective from members of the Pulmonary Pathology Society. Arch. Pathol. Lab. Med..

[18] Poletti V., Petrarulo S., Piciucchi S., Dubini A., De Grauw A.J., Sultani F., Martinello S., Gonunguntla H., Ravaglia C. (2024). EBUS-guided cryobiopsy in the diagnosis of thoracic disorders. Pulmonology.

[19] Salcedo Lobera E., Codeso F.M.P., Casado Miranda E. (2023). Endobronchial ultrasound-guided transbronchial mediastinal cryobiopsy: Series of 50 cases. Rev. Clín. Esp..

[20] Kamath S., Jahangir A., Daouk S., Youness H.A. (2025). Mediastinal lymph node cryobiopsy guided by endobronchial ultrasound: A comprehensive review of methods and outcomes. Mediastinum.

[21] Todisco F., Patrucco F., Veljkovic A., Vallese G.C., Indellicati D., Valsecchi L., Riberi L., Ielo G., Iovine P.R., Ubaldi M. (2025). Transbronchial Mediastinal Cryobiopsy Guided by Endobronchial Ultrasound in Addition to Endobronchial Ultrasound-Guided Transbronchial Needle Aspiration in the Diagnosis of Ilo-Mediastinal Lymphadenopathy Without Obvious Primary Lung Neoplastic Lesion: A Prospective Multicenter Study. J. Clin. Med..

[22] Ariza-Prota M., Pérez-Pallarés J., Fernández-Fernández A., García-Alfonso L., Cascón J.A., Torres-Rivas H., Fernández-Fernández L., Sánchez I., Gil M., García-Clemente M. (2023). Endobronchial ultrasound-guided transbronchial mediastinal cryobiopsy in the diagnosis of mediastinal lesions: Safety, feasibility and diagnostic yield—Experience in 50 cases. ERJ Open Res..

[23] Ariza-Prota M., Pérez-Pallarés J., Barisione E., Cruz-Rueda J.J., Onyancha S., Usturoi D., De Santis M., Salcedo-Lobera E., Ferrer-Pargada D., Corcione N. (2025). Enhancing diagnostic precision: A multicentric study of endobronchial ultrasound-guided transbronchial mediastinal cryobiopsy in lymphoproliferative disorders. ERJ Open Res..

[24] Cai Z., Yuan Y., Zhou Z., Ding H., Cheng Y., Liang L., Tuo Z., Zhou R. (2025). Factors influencing the diagnostic accuracy of lung cancer using endobronchial ultrasound-guided transbronchial needle aspiration. Am. J. Transl. Res..

[25] Deng M., Zheng Z., Zhang X., Xia Y., Tang F., Yang Z., Zhong C., Tong R., Zhou G., Li X. (2026). EBUS-guided transbronchial mediastinal cryobiopsy for diagnosing non-metastatic lymphadenopathy: A randomized controlled trial. Med.

[26] Beattie J., Nasim H., Chen J., Bharwani A., Lara J., Parrish R., Swenson K., Parikh M., Karlin K., A Vanderlaan P. (2026). Endobronchial ultrasound-guided sampling: Diagnostic yield of needle biopsy and cryobiopsy in addition to needle aspiration. Ann. Am. Thorac. Soc..

